# RACIAL/ETHNIC DISPARITY IN 4M-BASED PRIMARY CARE DELIVERY VIA TELEHEALTH IN PROVIDER SHORTAGE AREA

**DOI:** 10.1093/geroni/igac059.2226

**Published:** 2022-12-20

**Authors:** Rain Lee, Yi Hyun Kim, Laurie Kim, Ji Won Yoo, Andrea Maxi, Chelin Hong

**Affiliations:** Yeshiva University, New York, New York, United States; University of California, Berkeley, Berkeley, California, United States; University of Nevada, Las Vegas, Las Vegas, Nevada, United States; University of Nevada, Las Vegas, Las Vegas, Nevada, United States; Yeshiva University, New York, New York, United States; Monash University, San Diego, California, United States

## Abstract

**Background:**

Telehealth is a promising alternative to primary care delivery in provider shortage areas. The purpose of this study was to evaluate the implementation of the 4Ms (i.e., Medication, Mentation, Mobility, What Matters) framework in telehealth-based primary care in provider shortage areas by ethnic status.

**Methods:**

This study was a retrospective analysis of 184 older adults (60+) representing 5% of the total sample at urban primary care in ethnically and racially diverse populations. Data were retrieved from July 2020 to September 2021. 14 trained primary care providers participated in this study and provided the 4Ms as following: 1) Medication (e.g., deprescribe or reduce high-risk Medication); 2) Mentation (e.g., depression and cognition assessment with brief counseling); 3) Mobility (e.g., mobility and home safety assessments); 4) What matters (e.g., advance care planning). The current study measured components of the 4Ms per telehealth visit by ethnic/race status (white vs. non-white).

**Results:**

Overall, advance care planning (i.e., what matters) was the most discussed via telehealth (79%), followed by mobility (46.2%), Medication (16.8%), and Mentation (14.7%). To examine the disproportion of accessing telehealth by patients’ racial background, the independence test of chi-square showed that non-white populations were less likely to have access to telehealth than white patients (p = .02).

**Conclusion:**

There was an ethnic and racial disparity in the 4M framework application via telehealth in an urban primary care clinic. To sustain telehealth for patients in a healthcare shortage, ethnically and culturally specific training is needed, and linguistically diverse curricula are recommended.

